# Successful tricuspid valve repair and right atrial mass excision for calcified thrombus causing severe tricuspid regurgitation and near-occlusion of inferior vena cava

**DOI:** 10.1186/s13019-024-02571-8

**Published:** 2024-02-19

**Authors:** Caroline Pennacchio, Brad Rosinski, Richard Grimm, Shinya Unai

**Affiliations:** 1 Department of Thoracic and Cardiovascular Surgery, Heart, Vascular, and Thoracic Institute, Cleveland Clinic, 9500 Euclid Avenue, Cleveland, OH 44195 USA; 2 Department of Cardiovascular Medicine, Heart, Vascular, and Thoracic Institute, Cleveland Clinic, Cleveland, OH USA

**Keywords:** Right atrial thrombus, Right atrial mass, Tricuspid regurgitation

## Abstract

**Background:**

Calcified right atrial thrombus is rare and commonly occurs secondary to atrial fibrillation and long-term central venous catheterization which present risk for embolization. Treatment typically involves anticoagulation and antiplatelet therapy but rarely surgical excision can be performed, especially in patients with venous obstruction or concomitant valvular dysfunction.

**Case presentation:**

We present the case of a 69 year old symptomatic female with a history of atrial fibrillation and long-term venous catheterization found to have a large calcified right atrial thrombus causing inferior vena cava obstruction and severe tricuspid regurgitation. Patient underwent full median sternotomy with ascending arterial cannulation with superior vena cava and femoral venous cannulation. Intraoperatively, extensive right atrial calcified thrombus was found extending into the inferior vena cava and involving the septal portion of the tricuspid valve annulus causing regurgitation. The calcified thrombus was removed which resolved the inferior vena cava obstruction and the tricuspid valve was repaired by transecting septal leaflet chordae, commissuroplasty, and ring annuloplasty. Postoperative course was uncomplicated and pathology confirmed a calcified right atrial thrombus. At 6 month follow up, the patient was asymptomatic with echocardiogram showing no inferior vena cava stenosis and trivial tricuspid regurgitation.

**Conclusions:**

Surgical excision of calcified right atrial thrombus is rare and is often indicated for symptomatic patients with extensive involvement causing venous inflow obstruction or valvular dysfunction. Sufficient preoperative imaging and a multi-disciplinary approach are essential for accurate diagnosis to guide targeted treatment. When the tricuspid valve is involved, repair is preferred over replacement in this patient population given their propensity for calcification and thrombus formation which may result in an increased risk of early bioprosthetic valve degeneration or mechanical valve thrombosis.

## Background

Calcified right atrial thrombus is rare and typically occur secondary to atrial fibrillation or long-term central venous catheterization which present risk for embolization [1, 2]. Treatment can range from antiplatelet, anticoagulation, or thrombolytic therapy to invasive options such as surgical or percutaneous removal [3, 4]. Invasive excision can be necessary for accurate diagnosis and prevention of embolic events [5, 6]. However, extensive calcified right atrial thrombus can result in inferior vena cava occlusion [5] and involvement of the tricuspid valve may precipitate tricuspid regurgitation which would necessitate surgical repair [7].

## Case presentation

We present the case of a 69 year old female with history of atrial fibrillation and breast cancer status post mastectomy, radiation, and chemotherapy with worsening dyspnea on exertion found to have a calcified right atrial mass and severe tricuspid regurgitation likely secondary to eight-month duration of central venous access for chemotherapy twelve years prior.

Cross-sectional imaging displayed a calcified mass at the inferior vena cava/right atrial junction with extension into the intrahepatic portion of the inferior vena cava, and along the interatrial septum with additional calcifications of the right ventricle resulting in tethering of the septal leaflet of the tricuspid valve.

Transthoracic echocardiogram showed near-occlusion of the inferior vena cava and severe tricuspid regurgitation with a medially directed jet secondary to a restricted septal leaflet due to subvalvular calcium extending along the septum and involving the valve. A dilated right ventricle with normal right ventricular systolic function, and elevated right ventricular systolic pressure consistent with mild pulmonary hypertension were also observed. Given the patient’s age, coronary angiography was performed that did not identify coronary artery disease.

**Fig. 1:**
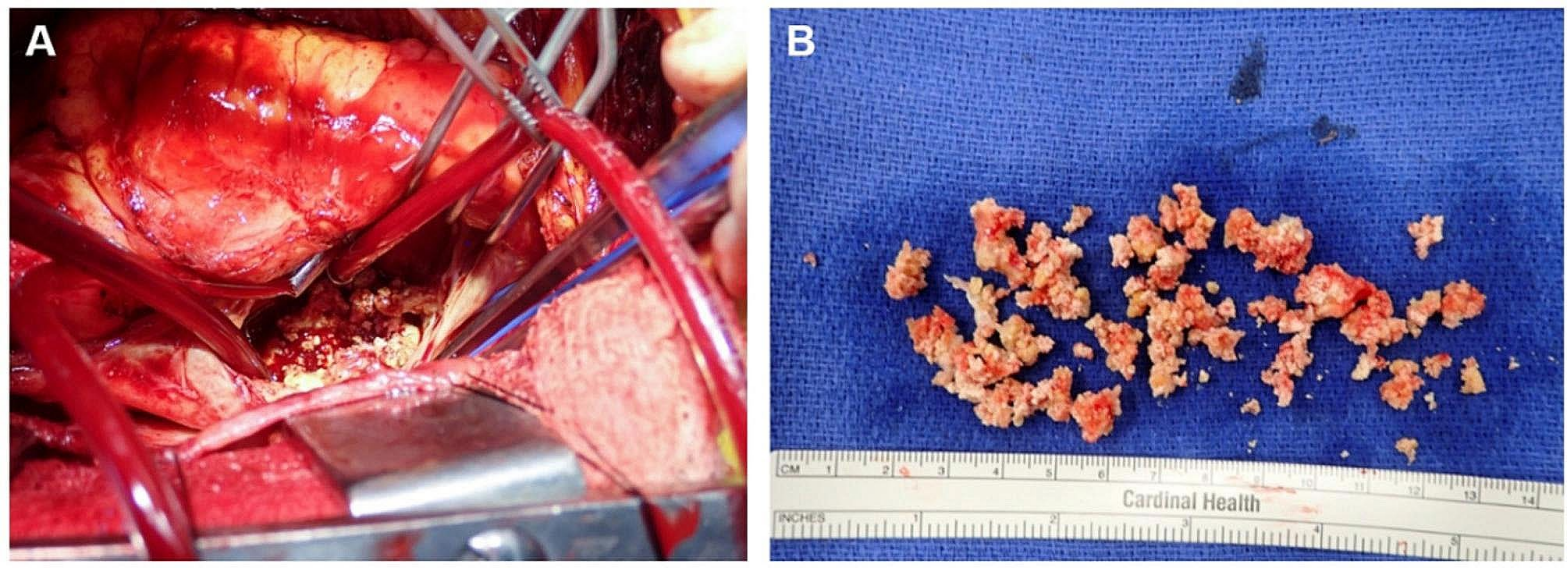
Intraoperative images of calcified right atrial thrombus. (**A**) Right atrial exposure. Calcified right atrial thrombus with extensive involvement of tricuspid valve and near occlusion of the inferior vena cava. (**B**) Gross depiction of excised right atrial calcified thrombus

Right femoral venous access was obtained and patient underwent full median sternotomy. The distal ascending aorta was cannulated with a 21 French cannula. Due to inferior vena cava partial occlusion, a 24 French cannula was advanced from the right femoral vein to the inferior vena cava under transesophageal echocardiogram guidance with the tip of the cannula placed just distal to the inferior vena cava/right atrial junction. A 24 French right angle cannula was inserted into the superior vena cava. Once cardiopulmonary bypass was initiated, the aorta was crossclamped, a single dose of Del Nido cardioplegia was delivered to the aortic root, and superior vena cava and inferior vena cava were snared. The patient was maintained at normothermia. The right atrium was opened via transverse incision. Upon inspection, there was a large amount of calcified thrombus within the right atrium extending into the inferior vena cava and involving the septal portion of the tricuspid valve annulus which was removed via sharp and blunt dissection (Fig. 1). Of note, the calcified mass did not have the appearance of endocarditis. The inferior vena cava cannula was then clamped for approximately five seconds to flush any debris. Attention was then turned to the tricuspid valve which showed severely restricted motion of the septal leaflet due to thickening and tethering of the associated chordae secondary to calcification extending from the right atrium to the right ventricle. Three chordae were then transected to improve coaptation. The tricuspid annulus was sized for a 26 mm partial annuloplasty ring. The ring was then sewn to the tricuspid annulus using horizontal mattress sutures and septal-anterior leaflet commisuroplasty was performed. The valve was tested and was found to be competent. The right atrium was closed in the standard fashion. The patient was weaned from cardiopulmonary bypass with a crossclamp time of 40 min and total cardiopulmonary bypass time of 68 min. Intraoperative transesophageal echocardiogram showed trivial tricuspid regurgitation and normal biventricular function. The chest was closed in the standard fashion and patient was transported to the intensive care unit in stable condition. Pathology confirmed calcified thrombus.

Postoperative course was unremarkable and she was discharged on postoperative day 9. At 6-month follow up, she was asymptomatic with significantly improved exercise tolerance. Echocardiogram at this time again demonstrated trivial tricuspid regurgitation without evidence of inferior vena cava stenosis or occlusion.

## Discussion/Conclusion

This case demonstrates the successful surgical treatment of calcified right atrial thrombus causing severe tricuspid regurgitation and near-occlusion of the inferior vena cava. Treatment of calcified right atrial thrombi has often involved the use of anticoagulation with surgical treatment reserved for the excision of large and/or mobile thrombi [1, 2, 8]. Sufficient cross-sectional imaging and a multi-disciplinary approach are essential for accurate diagnosis to guide targeted treatment. Surgical treatment is often necessary when the presence of the calcified thrombus results in venous obstruction and disrupts the function of the tricuspid valve. Venous cannulation should be tailored to the patient’s specific anatomy and right atrial calcium burden. When the tricuspid valve is involved, repair is preferred over replacement in this patient population given the propensity for calcification and thrombus formation and thus may result in an increased risk of early bioprosthetic valve degeneration or mechanical valve thrombosis.

## Data Availability

Not applicable.
